# Cerebral Cysticercosis Presenting With Recurrent Epileptic Seizures

**DOI:** 10.7759/cureus.8153

**Published:** 2020-05-16

**Authors:** Pham Hong Van, Nguyen Quang Thieu, Cao Ba Loi, Ngo Minh Xuan, Huynh Quang Huy

**Affiliations:** 1 General Planning Department, National Hospital of Traditional Medicine, Hanoi, VNM; 2 Parasitic Infectious Department, National Institute of Malariology/Parasitology and Entomology, Hanoi, VNM; 3 Pediatrics, Pham Ngoc Thach University of Medicine, Ho Chi Minh City, VNM; 4 Department of Radiology, Pham Ngoc Thach University of Medicine, Ho Chi Minh City, VNM

**Keywords:** cerebral cysticercosis, seizure, magnetic resonance imaging

## Abstract

Cerebral cysticercosis is the most common parasitic disease of the human nervous system. It is endemic to some tropical countries but has rarely been described in Vietnam. We report three cases of neurocysticercosis in patients from north-west Vietnam presenting with recurrent epileptic seizures. Hypereosinophilia and positive immunoglobulin G (IgG) antibodies to cysticercosis were detected in two patients and three patients, respectively. The brain MRI showed multiple ring-enhancing cerebral lesions with a well-defined border. Scolexes were demonstrated on fluid attenuation inversion recovery (FLAIR) sequence as small images associated with a hyperintense cyst wall. Treatment of cerebral cysticercosis infection with albendazole 15 mg/kg/day x 21 days along with antiepileptic drug therapy usually results in a favorable outcome. These results highlight that cerebral cysticercosis should be suspected in patients from an endemic area who present with headaches and/or epileptic seizures.

## Introduction

Cysticercosis, a type of tissue infection caused by the young form of the tapeworm, is the most common parasitic disease of the nervous system in humans [1]. Infected patients might show little or no clinical symptoms for years. A particular form termed cerebral cysticercosis that affects the brain might produce neurological symptoms, and this is one of the most frequent causes of seizures in developing countries [2]. Cerebral cysticercosis is the outcome of an invasion of the cerebral tissue by the intestinal tapeworm *Taenia solium* at the larval stage, which is found in pigs [3].

Clinical presentations are usually nonspecific and depend on the number, size of cysts, positions, as well as the adaptive immune response of the human body. Cysticercosis commonly affects the cortex and cerebral hemispheres. Cysticercosis is one of the most frequent tropical diseases encountered in Sub-Saharan Africa, South America, Southeast Asia, China, and India [4]. Cysticercosis is endemic to Vietnam, but there have been few studies with regard to the prevalence of the disease in the country. Moreover, little is known about the role of the cysticercosis as an epileptogenic lesion and a causal factor of headaches and epilepsy [5].

The word neurocysticercosis is generally used to refer to cysts in the brain parenchyma. It can cause non-specific symptoms such as headaches, nausea, and seizures [6]. Cysticerca in the parenchyma of the human brain is usually 5-20 mm in diameter. The lesions might be around 6 cm in diameter and are located in fissures and subarachnoid space. It may be life-threatening if there are multiple cysticerca. In this report, we present three cases with prolonged seizures due to cerebral cysticercosis that were diagnosed and treated at our center.

## Case presentation

The three male patients were admitted to the Vietnam National Institute of Malariology, Parasitology and Entomology in 2018 with a medical history of recurrent seizures for three years (case 1), four days (case 2), and seven years (case 3), respectively. All patients hailed from the northwest area of Vietnam. Two patients (cases 1 and 3) had headaches, and one patient (case 1) had nausea occasionally (Table 1). Two cases (cases 1 and 3) had been previously treated with antiepileptic drugs, but the seizures were not well controlled. Case 3 had not received any treatment at the time of hospital admission.

**Table 1:** Clinical features of the patients

Case	Age	Sex	Seizure	Headache	Nausea
1	44	Male	Yes	Yes	Yes
2	51	Male	Yes	No	No
3	49	Male	Yes	Yes	No

Neurological examination did not find any evidence of cognitive decline, intracranial hypertension, or focal neurological deficits. Laboratory investigation showed hypereosinophilia in two patients (cases 1 and 2), and positive immunoglobulin G (IgG) antibodies to cysticercosis in three patients (Table 2).

**Table 2:** Laboratory investigations of the patients ELISA: enzyme-linked immunosorbent assay; IgG: immunoglobulin G

Case	Red blood cells	White blood cells	Eosinophil	Serum ELISA test for cysticercosis
1	5.25 x 10^12^/L	8.8 x 10^9^/L	6.2%	Positive IgG (0.464)
2	5.1 x 10^12^/L	8.6 x 10^9^/L	6.0%	Positive IgG (0.562)
3	4.44 x 10^12^/L	12.5 x 10^9^/L	0.7%	Positive IgG (0.446)

**Figure 1:**
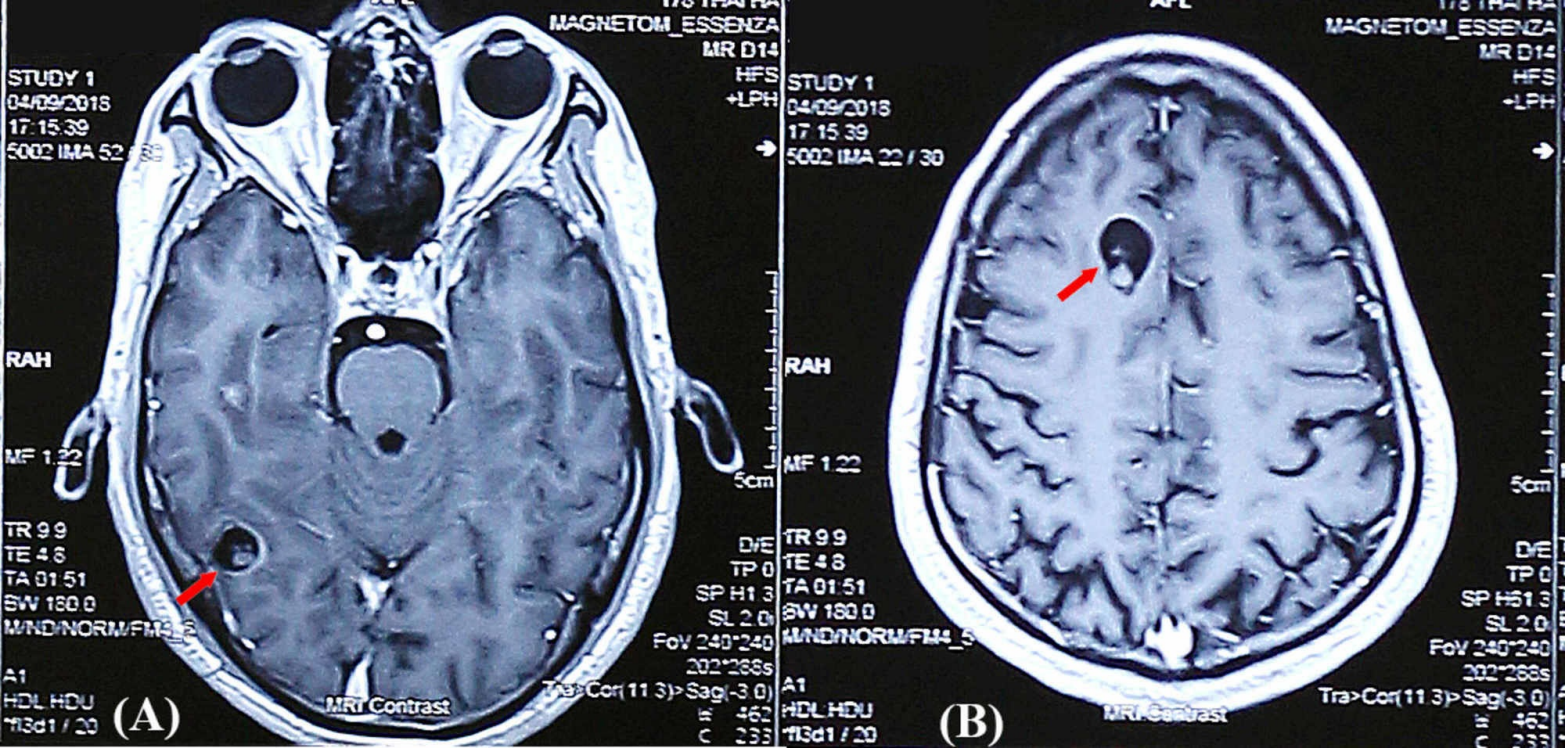
MRI findings of case 1 (axial T1 weighted images) The axial T1 weighted image after contrast showed a ring-enhancing cystic lesion with a well-defined border in the right occipital lobe. The diameter of this lesion was 11 mm. A scolex was demonstrated as a small image associated with a hyperintense cyst wall (A). A similar lesion was found in the right frontal lobe with 10 mm in diameter (B) MRI: magnetic resonance imaging

**Figure 2:**
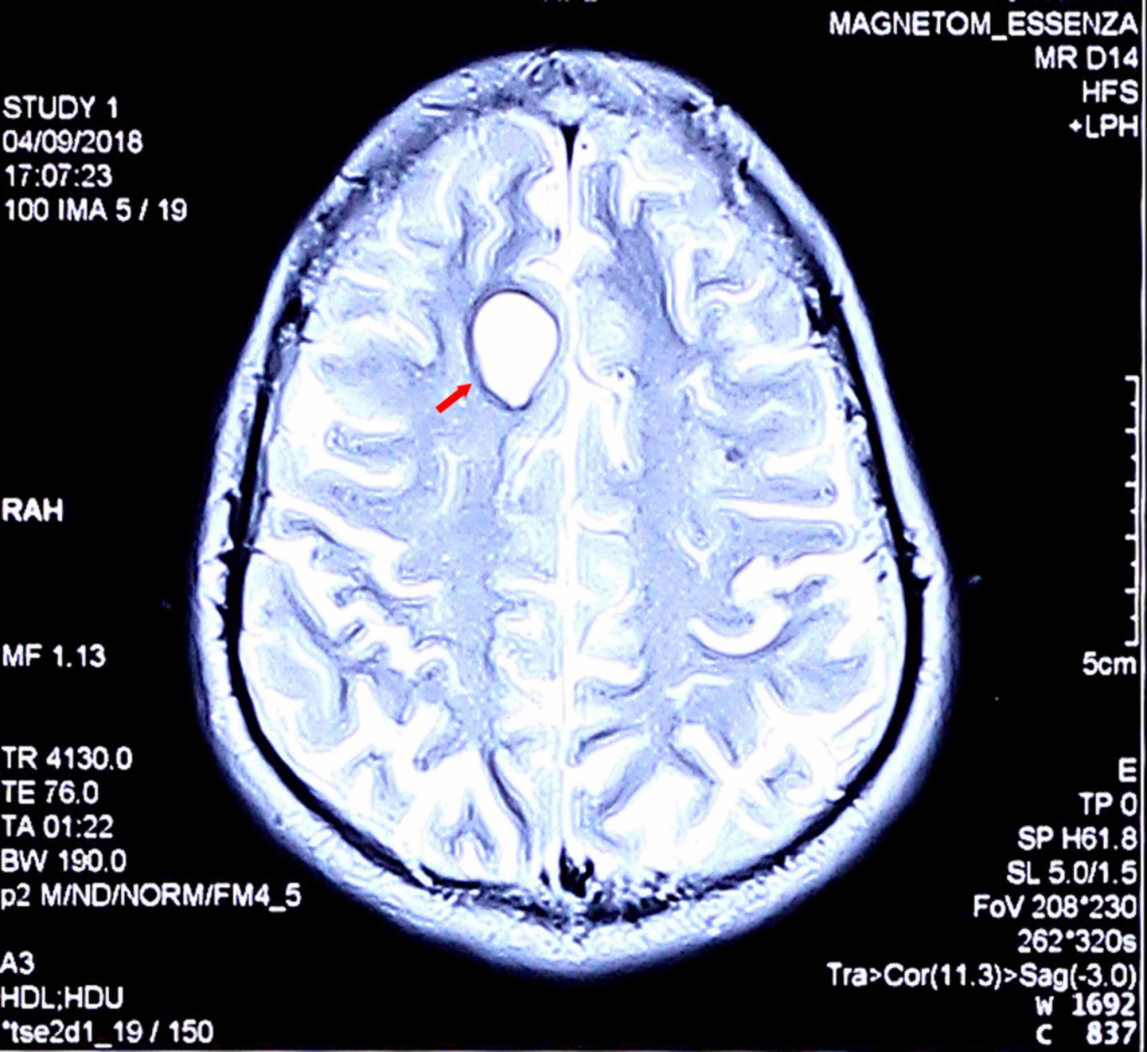
MRI axial T2 weighted image of case 1 The axial T2 weighted image showed a hyperintense, well-defined border cystic lesions in the right frontal lobe with a diameter of 11 mm MRI: magnetic resonance imaging

**Figure 3:**
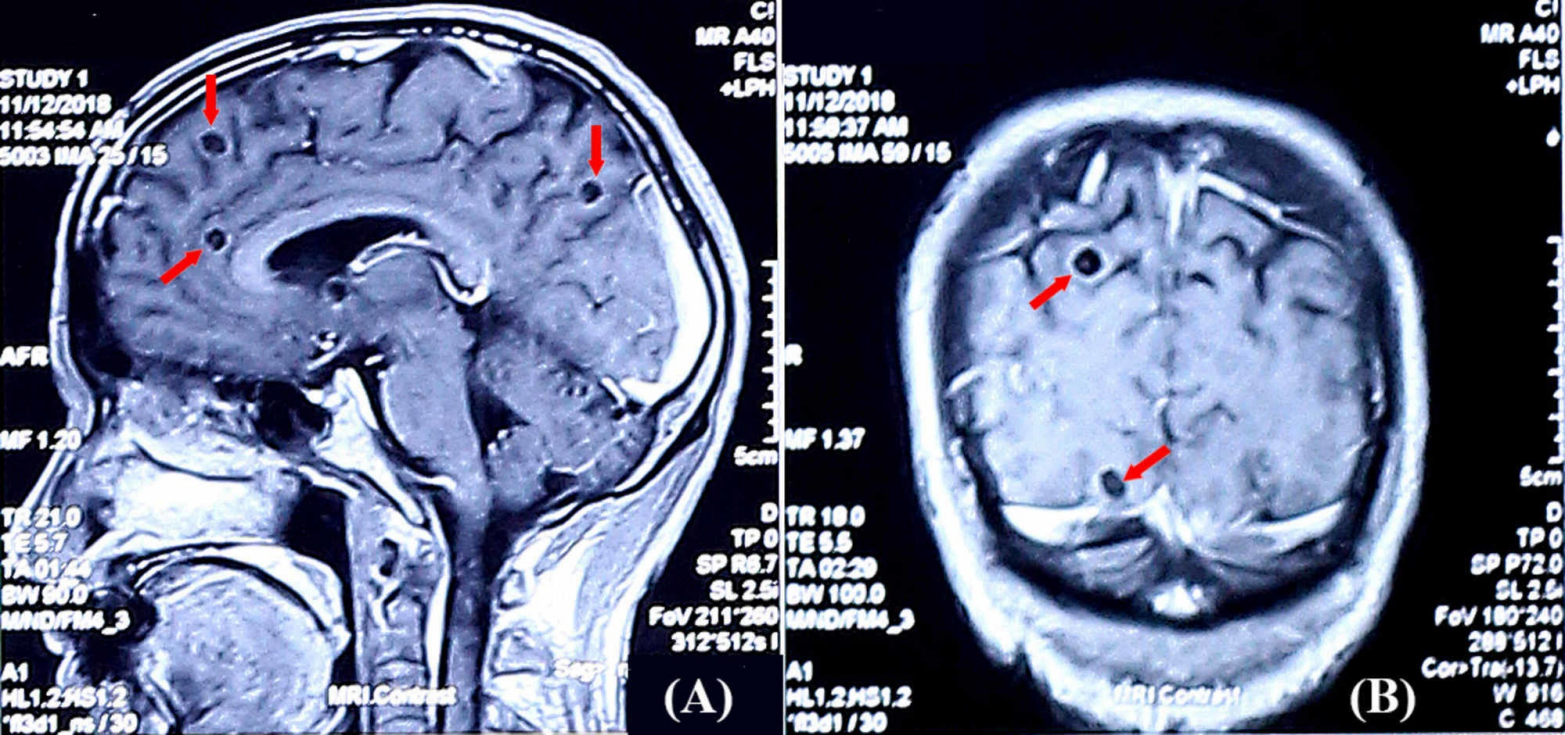
MRI findings of case 2 The sagittal T1 weighted image after contrast showed well-defined ring-enhancing cystic lesions (5-10 mm) in the cerebral hemisphere (A). The coronal T1 weighted image after contrast showed well-defined ring-enhancing cystic lesions (7-9 mm) in the cerebral hemisphere. Scolexes were demonstrated inside the lesions (B) MRI: magnetic resonance imaging

**Figure 4:**
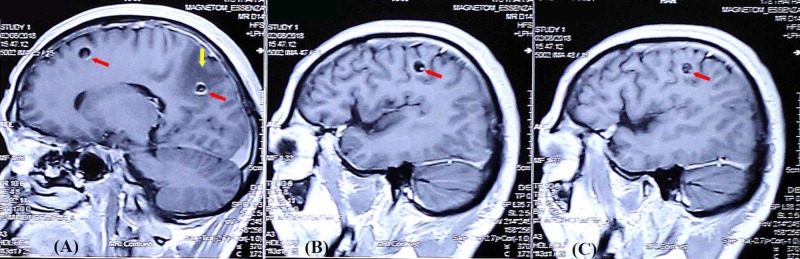
MRI findings of case 3 The sagittal T1 weighted image after contrast showed well-defined ring-enhancing cystic lesions (5-10 mm) in the right frontal lobe and parietal lobe with the scolex of *Taenia solium *cysticercosis inside (red arrows). The cystic lesion in the right temporal lobe had perilesional edema (yellow arrow) (A). A similar lesion located in the left parietal lobe was enhanced after contrast with a scolex of *Taenia solium* cysticercosis inside (red arrow) (B and C) MRI: magnetic resonance imaging

The brain MRI showed multiple ring-enhancing cystic lesions with well-defined border. A scolex was demonstrated as a small image associated with a hyperintense cyst wall (Figure 1, 2, 3, 4).

All three patients were diagnosed with cerebral cysticercosis, which was determined to the cause of their epilepsy, and then treated with albendazole (15 mg/kg/day x 21 days) and prednisolone (40 mg per day) along with antiepileptic drug therapy. No cerebral operation was performed. Follow-ups after three months, six months, and one year following discharge noted that all patients were seizure-free without headache, nausea, or other neurological disorders. Blood tests have been within normal ranges since the third month after treatment.

## Discussion

Cerebral cysticercosis is the most common parasitic disease of the central nervous system and has been known as an epileptogenic lesion worldwide [7]. This disease appears sporadically in developed countries. Cerebral cysticercosis remains on the World Health Organization's list of neglected tropical diseases. It is endemic to many developing countries, especially in poverty-stricken zones of the world such as Latin America and Sub-Saharan Africa [8]. Cysticercosis is endemic to Vietnam, but there have been few studies with regard to the prevalence of the disease in the country. Moreover, little is known about the role of the parasite as a causal agent of epilepsy and headaches [5]. All the patients in our series were male, which is consistent with the finding of other studies that cerebral cysticercosis is slightly more likely to be found in males than females [9,10].

The diagnosis of cerebral cysticercosis is challenging, and sometimes it is achievable only after the elimination of the disease. Clinical presentations are usually non-specific, depending on the number and position of cystic lesions in the human nervous system and the inflammation evoked by the parasites. The most common symptom is seizures, but chronic headaches, cognitive decline, intracranial hypertension, and focal neurological deficits are also frequently present. The final diagnosis is usually based on the correlation between clinical features and neuroimaging. This association may be considered highly specific and sensitive to rare diseases [11]. A patient with a well-defined ring-enhancing lesion on MRI should be suspected of having cysticercosis, tuberculoma, or sparganosis [12].

A diagnosis of cysticercosis can be made on MRI when the cystic lesions appear hypointense on T1 weighted image, hyperintense on T2 weighted image, with peripheral enhancement. Edema with variable degrees may be seen in the tissues surrounding the lesions [13,14]. Our patients were diagnosed with cerebral cysticercosis, mainly based on brain MRI findings and serological antigen detection. Clinical features such as recurrent seizures, occasional headaches, and nausea were non-specific for cerebral cysticercosis. Focal neurological deficits were not found in any of the patients.

Considering the nature of the interactions that exist between these two conditions, a number of hypotheses can be considered. Firstly, it has been suggested that cerebral cysticercosis is a direct cause of epilepsy. This theory would need to consider the dispute over whether direct provocation by inflammation of and structural damage to the brain parenchyma would actually constitute epilepsy versus acute symptomatic seizures. Secondly, it is postulated that cerebral cysticercosis is a component of the epileptogenic pathway, contributing to the development of the disease, but not acting directly as a causative factor [15]. Cerebral cysticercosis could be considered an initial precipitating injury for epilepsy, where cerebral cysticercosis infection acts as a trigger for future spontaneous recurrent seizures and the development of an epileptogenic profile but is not directly responsible for these subsequent individual seizure events. Thirdly, it is posited that there is an indirect link between epilepsy and cerebral cysticercosis due to an independent factor, causing a misleading association between the two conditions. Some studies have shown high rates of familial aggregation of cerebral cysticercosis, indicating the presence of a genetic predisposition to the disease, which may also be linked to increased risk of epilepsy [16]. Finally, there exists a theory that the high rates of comorbidity of cerebral cysticercosis and epilepsy are coincidental due to high concurrent prevalence rates of the two conditions, which occur independently from one another, in certain countries [17,18].

## Conclusions

Our results from this case series highlight that cerebral cysticercosis should be suspected in patients hailing from an endemic area who present with epileptic seizures and headaches. Treatment of cysticercosis infection with appropriate medications, along with antiepileptic drug therapy, usually results in favorable outcomes.
